# Mechanoregulation of Vascular Endothelial Growth Factor Receptor 2 in Angiogenesis

**DOI:** 10.3389/fcvm.2021.804934

**Published:** 2022-01-11

**Authors:** Bronte Miller, Mary Kathryn Sewell-Loftin

**Affiliations:** ^1^Department of Biomedical Engineering, University of Alabama at Birmingham, Birmingham, AL, United States; ^2^O'Neal Comprehensive Cancer Center, University of Alabama at Birmingham, Birmingham, AL, United States

**Keywords:** angiogenesis, VEGFR-2, mechanobiology, mechanoregulation, endothelial cells

## Abstract

The endothelial cells that compose the vascular system in the body display a wide range of mechanotransductive behaviors and responses to biomechanical stimuli, which act in concert to control overall blood vessel structure and function. Such mechanosensitive activities allow blood vessels to constrict, dilate, grow, or remodel as needed during development as well as normal physiological functions, and the same processes can be dysregulated in various disease states. Mechanotransduction represents cellular responses to mechanical forces, translating such factors into chemical or electrical signals which alter the activation of various cell signaling pathways. Understanding how biomechanical forces drive vascular growth in healthy and diseased tissues could create new therapeutic strategies that would either enhance or halt these processes to assist with treatments of different diseases. In the cardiovascular system, new blood vessel formation from preexisting vasculature, in a process known as angiogenesis, is driven by vascular endothelial growth factor (VEGF) binding to VEGF receptor 2 (VEGFR-2) which promotes blood vessel development. However, physical forces such as shear stress, matrix stiffness, and interstitial flow are also major drivers and effectors of angiogenesis, and new research suggests that mechanical forces may regulate VEGFR-2 phosphorylation. In fact, VEGFR-2 activation has been linked to known mechanobiological agents including ERK/MAPK, c-Src, Rho/ROCK, and YAP/TAZ. In vascular disease states, endothelial cells can be subjected to altered mechanical stimuli which affect the pathways that control angiogenesis. Both normalizing and arresting angiogenesis associated with tumor growth have been strategies for anti-cancer treatments. In the field of regenerative medicine, harnessing biomechanical regulation of angiogenesis could enhance vascularization strategies for treating a variety of cardiovascular diseases, including ischemia or permit development of novel tissue engineering scaffolds. This review will focus on the impact of VEGFR-2 mechanosignaling in endothelial cells (ECs) and its interaction with other mechanotransductive pathways, as well as presenting a discussion on the relationship between VEGFR-2 activation and biomechanical forces in the extracellular matrix (ECM) that can help treat diseases with dysfunctional vascular growth.

## Introduction

Angiogenesis, or the development of blood vessels from preexisting vasculature, is a pillar of normal development and growth; however, various disease states are associated with dysregulation of blood vessel growth. For example, tumors express irregular and tortuous vasculature, while ischemia is the lack of functional vessels, creating hypoxic environments and ultimately leading to tissue death. Much research has focused on either inhibiting or promoting angiogenic processes to treat diseases, but these therapeutic approaches have shown mixed results. Perhaps some of these failures are due in part to the focus on biochemical regulation of angiogenesis while ignoring mechanical cues that could also be controlling blood vessel development. For this review, we will use biochemical signaling to refer to the binding of vascular endothelial growth factor (VEGF) to VEGF receptor 2 (VEGFR-2), while biomechanical signaling will refer to VEGFR-2 activation or downstream signaling induced by non-ligand binding cues.

**Figure 1:**
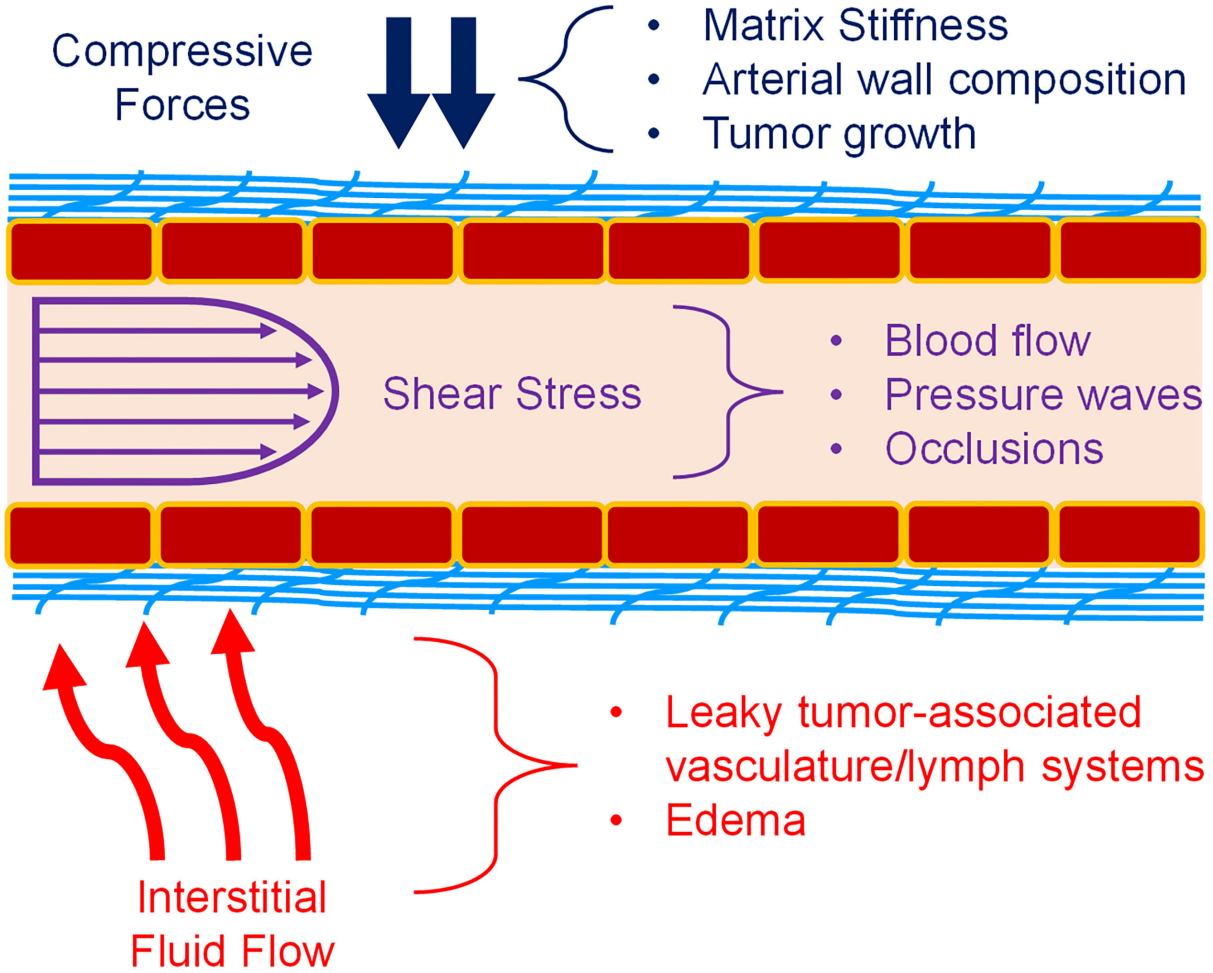
Mechanical forces in blood vessels. Vascular ECs (red boxes) experience a variety of forces. Compressive forces (blue) can be caused by matrix (light blue) stiffness, tumor expansion, and surrounding cells. Shear stress (purple) caused by blood flow is also a major component of the vascular environment. Pulsatile flow and atherosclerosis also affect fluid flow and the resulting shear stress. Interstitial fluid flow and pressure (red) is also present during angiogenesis, and especially in the TME. Permeable vasculature is commonly present in disease states such as tumors and edema. Figure was derived based on the following references: (1–13).

Mechanotransduction is the process by which mechanical signals from the extracellular matrix (ECM), nearby cells, or surrounding fluids interact with a cell and are subsequently processed into a biochemical signaling cascade of various proteins to regulate the cellular response to the mechanical cue. In the vasculature, the endothelial cells (ECs) that comprise the vessel walls are in a constant state of mechanical stimulation, through a variety of forces (Figure 1). This includes not only shear stress from blood flow within the vessels, but also interstitial flow over the vessels from the surrounding tissues (1–3). There are compressive strains due to matrix composition or stiffness, as well as regulation of the vascular tone by vascular smooth muscle cells (VSMCs) or pericytes (4, 5). Also, the geometry of blood vessels induces curvature stresses in the endothelial cells (1, 3, 5). Finally, in larger vessels of the arterial system, a pulsatile fluid pressure wave is experienced with every heartbeat (3). Together, these forces may alter endothelial cell behaviors in underappreciated manners that could affect the growth and development of new vasculature. During development, these forces guide cell activity, but many disease states possess an altered biomechanical milieu that dysregulates the vasculature. For example, in the tumor microenvironment (TME), matrix remodeling initiated by stromal and tumor cells increases matrix stiffness, usually through increased collagen deposition and organization. This increases both compressive forces on the cells and vessels, forcing higher levels of interstitial flow as fluids are “squeezed” from the vessels and generating irregular shear stresses. These types of changes have been associated with the regulation of tumor-associated angiogenesis, as will be discussed below. Additionally, arterial stiffening, which is common with increased age, is due to changes in elastin and collagen content of the arterial wall. These changes alter forces induced by blood flow and could significantly impact the endothelial cell signaling processes. Understanding how mechanotransduction by endothelial cells regulates angiogenesis will provide critical knowledge in how to promote or inhibit blood vessel growth in the dysregulated biomechanical environments of various disease states.

The ligand VEGF and one of its receptors VEGFR-2 are the major regulators of angiogenesis; however, VEGFR-2 has been known to be activated independent of its ligand (14, 15). Several studies have demonstrated that mechanical forces can regulate VEGFR-2 expression and activation (15–21). For example, ECs exposed to shear stress showed formation of a VEGFR-2 and VE-cadherin-β-catenin complex that acted as a mechanotransducer and allowed the cells to activate downstream pathways such as p38 and Akt (22). Since VEGFR-2 can promote angiogenesis, changes in the physical environment could inhibit or promote blood vessel development. This paper will focus on how VEGFR-2 on ECs has been shown to be mechanically-activated, which downstream mechanotransductive factors are regulated by VEGFR-2 signaling, and how knowledge and future studies of this phenomenon could help develop treatments for various disease states.

## VEGFR-2

**Figure 2:**
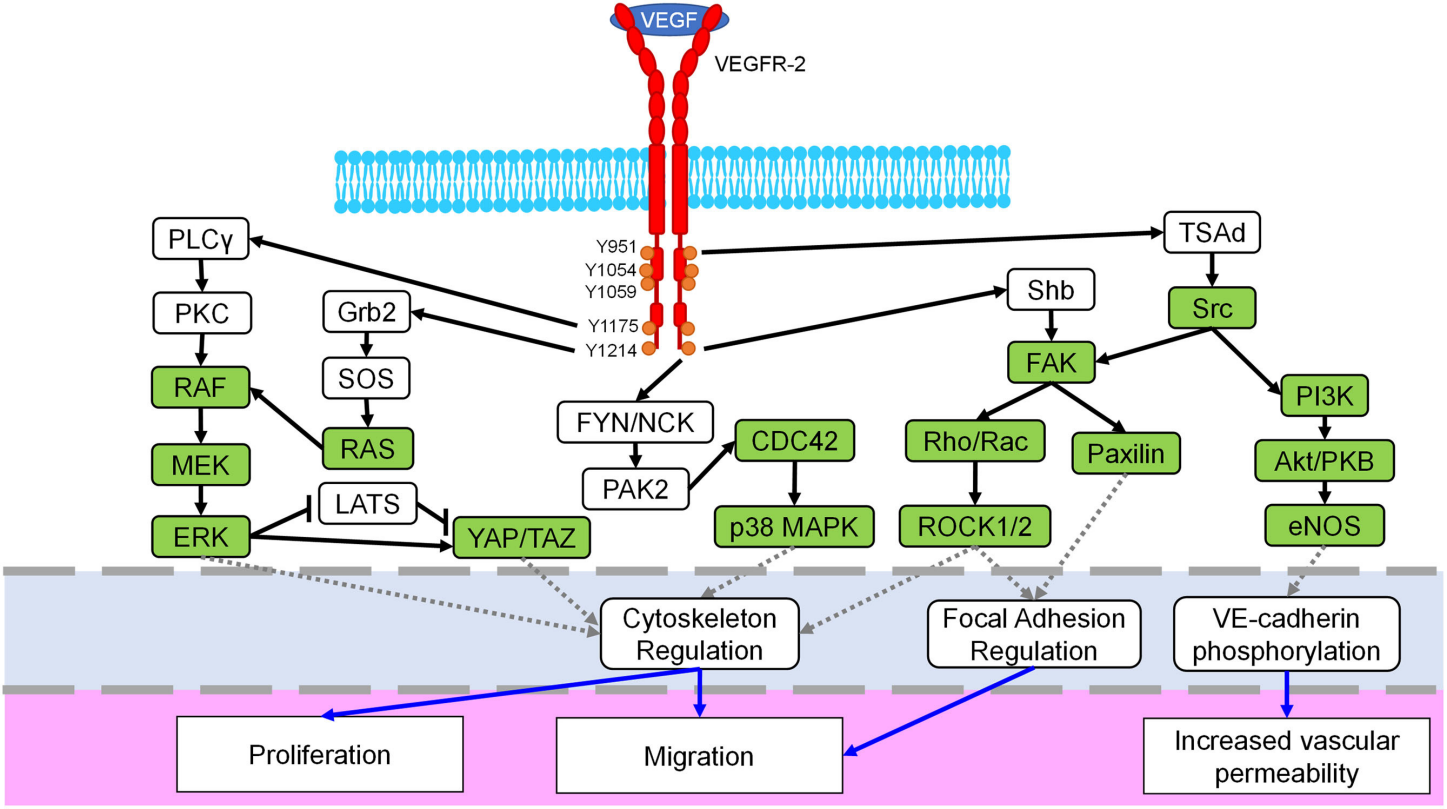
Mechanosignaling in the VEGFR-2 pathway. The VEGFR-2 receptor (red) dimerizes when bound by the VEGF ligand, spans the cell membrane (blue) and possesses multiple phosphorylation sites in the intracellular domain (orange). Downstream signaling initiated by VEGFR-2 includes several known mechanotransductive proteins and enzymes (green) including the MEK/ERK, Rho/ROCK, Src, and YAP/TAZ pathways. Crosstalk between related pathways, i.e., ERK/p38, are not shown for the sake of clarity. The primary target of each pathway is identified with a dashed gray line (light blue region) and includes cytoskeletal regulation, focal adhesion regulation, and VE-cadherin signaling. Finally, resulting cellular phenotypes for each mechanosignaling pathway are indicated with a solid blue arrow in the lowest region of the figure. Figure was derived based on the following references: (23, 24, 32–38).

Considered one of the key components of endothelial proliferation and vascular growth, VEGFR-2, also known as fetal liver kinase 1 (Flk-1) or kinase insert domain receptor (KDR), is mostly found within vascular ECs, though it is also weakly expressed in other cells such as osteoblasts, hematopoietic cells, and megakaryocytes (23–26). Although this receptor tyrosine kinase (RTK) is primarily known for its angiogenic signaling effects, it is also involved in embryonic development, differentiation, and EC migration (27–29). Regulation of these processes is controlled by additional signaling pathways and kinase activity including Rho/ROCK, ERK, and Src (30, 31). In fact, many Rho-GTPases and mechanotransductive transcription factors are downstream of VEGF binding to VEGFR-2 (Figure 2). There are three domains of VEGFR-2: extracellular, transmembrane, and intracellular. Ligands bind to the extracellular region, which is rich in immunoglobulin domains, and the transmembrane domain stabilizes receptor dimerization (39, 40). The intracellular region contains a kinase domain and several tyrosine residues, which permit VEGFR-2 to act as an RTK and activate a variety of signaling cascades within ECs (32). Some of the prominent phosphorylation locations are Y1054/Y1059 which are necessary for angiogenic activation (41). The Y951 residue is more specific, causing Src activation and VE-cadherin phosphorylation; these effects lead to increased vascular permeability and mitogenesis (42, 43). Another well-studied phosphorylation site is Y1175, which interacts with Shb and activates PLCγ/MAPK, promoting migration and proliferation (44–46). Furthermore, Y1214 is a prominent site commonly associated with activation of p38, cdc42, Akt, and ERK pathways (33, 47). There are seven members of the VEGF ligand family: placenta growth factor (PIGF), four mammalian VEGFs (VEGF-A, VEGF-B, VEGF-C, VEGF-D), viral VEGF-E, and VEGF-F from snake venom (23). Each of these members of the VEGF ligand family has splice variant isoforms of varying molecular weights, with VEGF-A isoforms being the most prominent in directing blood vessel growth (23, 34, 48). VEGFR-2 dimerizes when activated, and though it can bind to other VEGF receptor family members, VEGFR-1 and VEGFR-3, to control migration and lymphangiogenesis, a VEGFR-2 homodimer is the primary regulator of blood vessel development (32, 39, 49–51). When inactive, VEGFR-2 colocalizes with caveolin-1, which resides in caveolae of the plasma membrane and is involved in cell signaling roles such as negatively regulating VEGFR-2 (52). However, once activated VEGFR-2 dissociates from caveolin-1, and VEGFR-2 is subsequently phosphorylated, endocytosed, and degraded (35, 52, 53). This phosphorylation and internalization of VEGFR-2 then leads to the activation of various signaling pathways, causing different reactions such as increased vascular permeability due to VE-cadherin endocytosis (54).

Two other members of the VEGF receptor family are VEGFR-1 and VEGFR-3, which also bind members from the VEGF family (34). Neuropilin-1 (NRP-1) and neuropilin-2 (NRP-2) act as co-receptors to members of the VEGF-receptor family and have been shown to play roles in vascular growth in developmental angiogenesis as well as tumor-associated angiogenesis (23, 55, 56). Similar to VEGFR-2, VEGFR-1 also regulates angiogenesis, specifically by inhibiting pro-angiogenic signals due to binding kinetics with the VEGF ligand. The extracellular domain of VEGFR-1 has higher affinity for VEGF than VEGFR-2, and evidence suggests this binding is responsible for angiogenic inhibition; however, VEGFR-2 has a higher level of tyrosine kinase activity, resulting in the more prominent role in angiogenesis (23, 32, 34). Alternative splicing can form a soluble VEGFR-1 (sVEGFR-1) that contains no transmembrane or intracellular domain, and this variant has been shown to block proliferation of vascular ECs (57). On the other hand, VEGFR-3 is commonly associated with the development of the lymphatic system, or lymphangiogenesis, though it has a role in regulating other angiogenic properties such as VEGFR-2 expression (58–60). Similar to VEGFR-2, VEGFR-3 can be mechanically-activated by sheer stress, and it complexes with VEGFR-2 and VE-cadherin on the cellular membrane (61). Further discussion of the other VEGF receptors can be found in the following reviews: Melincovici et al. as well as Koch and Claesson-Welsh (23, 32).

## Angiogenesis

Angiogenesis is the growth of new blood vessels from preexisting vasculature, as opposed to vasculogenesis, which is the development of vessels *de novo* (62). Both angiogenesis and vasculogenesis have been shown to play roles in progression of diseases including cancer, ischemia, and arterial stiffening (63, 64). The process of angiogenesis can be classified as either intussusceptive, also known as splitting, or sprouting angiogenesis. Splitting angiogenesis is less studied and occurs when one vessel develops into two parallel vessels through the growth and fusion of tissue pillars in the middle of the original capillary or vessel (63, 65). Sprouting angiogenesis is more widely understood and involves nascent vessels entering a previously un-vascularized tissue region, which is common in embryonic development and certain disease states including the TME (66–68). Sprouting angiogenesis begins with a stimulus, such as hypoxia which affects oxygen sensors including hypoxia inducible factors (HIF), causing cancer or stromal cells to produce VEGF that diffuses to a preexisting vessel before binding VEGFR-2 (69, 70). The binding of VEGF to VEGFR-2 causes a breakdown of the basement membrane, and these ECs undergo a phenotypic shift into tip cells, with increased cell-matrix interactions, matrix remodeling, and high migratory potential. When a tip cell is formed, the VEGFR-2 receptors on filopodia follow the VEGF gradient, or other pro-angiogenic stimulus, to lead the growth of the sprouting blood vessel. As these cells migrate toward the angiogenic signal, stalk cells behind them proliferate and form the new vascular structure (63, 66, 69, 71). This basic understanding of angiogenesis is incomplete, however, because other biomechanical forces can also induce and direct vasculature development. For example, the highly invasive breast cancer cell line MDA-MB-231 migrates against flow, and increased matrix stiffness can decrease the EC vascular sprouting (72–75). Moreover, recent work has shown that angiogenesis can be driven through biomechanical strains induced in a matrix by cancer-associated fibroblasts (CAFs) or through mechanical manipulation with a magnetic bead system, in a manner that is not dependent on secreted VEGF (76, 77). Physical, and not solely chemical, signals in the ECM can result in altered angiogenic patterns, and such changes in physical or biomechanical signals are commonly seen in many disease states. As the role of biochemical regulation of the VEGFR-2 pathway is still only partially understood, the objective of this review is to describe key themes in VEGFR-2 mechanoactivation and mechanotransduction and how these are present in various pathological conditions.

### Angiogenesis in Disease

Dysregulation of angiogenesis occurs in various pathologies, and often this is caused or amplified by changes in the ECM. One common disease state is arterial stiffening which becomes more prevalent with age; this stiffening is also observed in several of the diseases discussed below, including peripheral arterial disease and ischemia (64). Late-passage or senescent ECs are more spread out and have decreased proliferative and angiogenic behaviors, with an increased chance to become apoptotic (78, 79). Understanding why this behavioral shift is associated with age could help to treat or even prevent further cardiovascular complications (79). In vascular walls, elastin and collagen help provide flexibility and rigidity, respectively; however, as an individual ages, concentration of elastin decreases and collagen increases (6, 7). As a result, the ECs of the blood vessel walls experience increased matrix stiffness and a higher arterial pulse pressure (80, 81). Furthermore, stiffer blood vessels can have increased permeability and become more likely to develop plaque accumulation, which can lead to further complications (82). Transcription factors YAP/TAZ, which reside downstream of VEGF/VEGFR-2, are upregulated on stiffer matrices and in disturbed flow, and *in vivo* inhibition of YAP/TAZ caused decreased atherosclerosis and EC inflammation (83, 84). Since many cells are known to respond to environmental mechanical cues, the natural stiffening of arteries could be a contributing factor of further cardiovascular diseases that are more common with age. Understanding how angiogenesis is affected by these physical changes would help develop new treatments that would target an overall cause of these illnesses instead of effects of arterial stiffening.

Another major disease state that involves angiogenesis is cancer progression, which has elucidated how the mechanical environment can affect blood vessel growth. When tumors are small, they can rely on simple diffusion for delivery of nutrients including oxygen; however, as the tumor increases in size, vasculature is required to provide cells with the increased nutrients for the increased cell numbers (2, 85). The TME is significantly different than normal, healthy tissue in both chemical and mechanical factors. First, there is an increase in proangiogenic factors such as VEGF within tumors (2, 75). Regarding mechanical components, tumor matrices are stiffer due to higher collagen production from stromal cells, and these collagen levels have been linked to Rho/ROCK and FAK regulation (8–11). New blood vessels grown during tumor progression are often not fully inter-connected with each other or the existing vasculature and can feature poor cell-cell contacts, leading to increased permeability or leakiness (12, 13). Furthermore, the interstitial fluid pressure is higher within tumors due to vessel compression leading to leaky, tortuous structures (12, 13). The combination of chemical and mechanical changes in the TME causes changes in angiogenic activity; understanding how such changes affect tumor growth and metastasis will help predict and treat this disease.

Unlike in the TME where angiogenesis is upregulated, some diseases are caused by or related to inhibited blood vessel growth, function, or survival. For example, peripheral arterial disease (PAD) causes hypoxic conditions in extremities often as a result of arterial blockage limiting blood flow. Late stages of this disease can lead to critical limb ischemia and eventually the need for limb amputation. Those that have PAD are at a higher risk of stroke and myocardial infarction, and many suffer from claudication, or pain from insufficient blood supply (86, 87). Mechanical changes such as disrupted fluid shear stress and stiffer arteries can result from plaque build-up and vessel narrowing present in many PAD cases (88–90). Also, patients suffering from PAD have been shown to have higher blood viscosity, especially when they experience regular claudication (91). Patients with PAD also show elevated levels of VEGF and lower levels of VEGFR-2 (92). Most current treatments focus on improving general cardiovascular health, with more drastic procedures such as stents being used if the condition worsens. Research is being done to determine if pro-angiogenic factors such as VEGF and fibroblast growth factor (FGF) could promote revascularization; however, these trials have shown limited success, suggesting that the angiogenic mechanisms of PAD are not fully understood (86, 93, 94). Since mechanical changes are present in the PAD disease state, how those changes affect the growth and deterioration of vasculature should be examined to produce more effective treatments aimed at revascularizing affected areas.

Related to PAD, ischemia occurs when tissue does not receive sufficient nutrients and oxygen due to insufficient or inefficient vasculature, creating a hypoxic environment and eventually leading to tissue death (95). Ischemia is classified based on the affected tissues. For example, myocardial ischemia is where cardiac tissue does not receive sufficient oxygen which can lead to multiple adverse events including arrythmias or myocardial infarctions (96). Myocardial infarctions cause heart tissue damage and scarring, with a higher amount of collagen deposited by cardiac fibroblasts during post-infarction events (97). This results in a stiffer matrix which is shown to express upregulated VEGF levels (98). The increased contractility of the myocardium, due to increased stiffness in the scar region, prevents revascularization of the affected area and severely impacts overall cardiac performance (99). Limb ischemia occurs when blood flow is limited to extremities such as the hands and legs. Both types of ischemia can be caused by atherosclerosis, or a build-up of plaque in the arteries, and limb ischemia can also be a complication of other diseases such as diabetes or PAD (96, 100, 101). Studies have shown upregulation of HIF, VEGF, and VEGFR-2 in limb ischemia, especially in acute limb ischemia as opposed to chronic (102, 103). Since limb ischemia is so similar to PAD, many of the mechanical changes present in PAD are also in ischemia, such as increased arterial stiffening and increased blood pressure (104). Studies have explored whether pro-angiogenic factors could promote vascularization in ischemic tissues, although so far these strategies have demonstrated mixed success, with single factors appearing to be less effective than combined treatments (93, 105, 106). A broader understanding of the relationship between angiogenic cytokines, VEGFR-2 mechanoactivation and mechanosignaling, and vessel reperfusion could benefit the development of this potential treatment strategy.

### Angiogenesis in Development

The cardiovascular system of the developing fetus is the first functional system generated, occurring even before circulation is established. Since an early embryo does not have any blood vessels, vasculogenesis must initially occur before angiogenesis later in development (62). VEGF and VEGFR-2 are both necessary in vasculogenesis and angiogenesis, with higher VEGF expression levels present during the former and lower expression during the latter (107, 108). Expression of VEGFR-2 is also upregulated early in development, especially after FGF is secreted (109). Knockdowns of VEGFR-2 and VEGF are embryonically lethal, demonstrating their significant roles in development (27, 110, 111). During development, cells naturally produce mechanical forces which can influence many factors including differentiation, migration, and angiogenesis as the tissues of the fetus form. These rapid changes in organization cause cells to experience stretch, pressure, and shear stress, which cause mechanoactivation of various proteins, such as cadherins, caveolins, Rho, and cdc42, and aid in development in normal physiological conditions (4). However, deletion of *vegfr-2* in a genetically-modified mouse model is embryonically lethal by stage E10.5 and showed no vasculature, suggesting that VEGFR-2 is required for vasculogenesis in development (27). Studying how these dynamic forces, and what additional cell types or matrix changes cause them, alter VEGFR-2 signaling could provide information about congenital vascular diseases and strategies for controlling blood vessel growth.

### Angiogenesis in Tissue Engineering

While there has been a significant rise in interest for tissue engineered scaffolds for replacement of disease tissues or organs in recent years, generation of large-scale systems has been limited by size restrictions due to limited nutrient diffusion. Currently tissue scaffolds with cells can be no more than approximately 200 μm away from a capillary, which represents the limit of oxygen diffusion in these tissue mimics (112, 113). Although many tissue engineering strategies have attempted to incorporate or promote vascularization during scaffold generation, there have been mixed results for creating functional and mature blood vessels, often because of the vascularization of the new tissue can take weeks (114, 115). Many studies have used pro-angiogenic factors, such as VEGF and FGF, to promote blood vessel growth. However, the concentrations and gradients of these factors is complex in native tissues, and imprecise use can limit vessel maturity, function, permeability, and structure (113, 115, 116). In addition to incorporating vascular growth factors, several groups have attempted to use biomechanical stimulation to prime these engineered scaffolds to generate more physiologically-relevant tissue functionality (117, 118). Understanding the unique ways in which biomechanical forces regulate blood vessel growth and vascular function through mechanosignaling related to the VEGFR-2 pathway could provide alternative strategies for developing larger tissues or even organs with a fully functional and interconnected vascular network.

### Stromal Cells

Other cells in the perivascular matrix can also act as a source of biomechanical signals to the ECs of the vasculature, potentially impacting the VEGFR-2 signaling axis. While some of these stromal cells, including vascular smooth muscle cells (VSMCs), weakly express VEGFR-2 and can play a role in ischemic conditions, the VEGFR-2 expression levels in ECs are significantly higher (23, 119–121). This includes VSMCs and pericytes in normal vasculature and stromal cells including cancer-associated fibroblasts (CAFs) in the tumor microenvironment. VSMCs comprise a concentric layer around the vascular lumen formed by endothelial cells in the tunica media and are most typically found on larger arterial vessels and veins; the overall function of these cells is to facilitate vasodilation or vasoconstriction based on physiological cues from the nervous system and driven by somatic need (122). These cells typically express alpha-smooth muscle actin and smooth muscle myosin heavy chain and can play a significant role in cardiovascular diseases including hypertension, ischemia, and atherosclerosis (123). Through regulation of vascular tone, VSMCs can directly lead to increased compressive forces on ECs in blood vessels as well as increases in shear stresses in the vessel lumen. Additionally, VSMCs demonstrate VEGFR-2 expression when exposed to hypoxic conditions (124). An additional example of mechanosensors present on both ECs and VSMCs is Piezo1, which is a non-selective cation channel that controls cellular response to shear stresses (125, 126). Pericytes, on the other hand, act as support cells in smaller vascular networks, specifically in capillary beds (122). There are multiple markers for pericytes including alpha smooth muscle actin, NG2 proteoglycan, platelet derived growth factor receptor beta, N-cadherin, and CD106 (127–129). Pericytes have been shown to directly interact with ECs in growing vascular networks, relying on heterotopic Notch signaling as well as heterotypic cadherin bonds to control sprouting angiogenesis and cell migration (130–132). These studies examined pericyte function and behavior in a variety of *in vitro* and *ex vivo* model systems in both cardiovascular development and disease as well as cancer progression. Moreover, expression of VEGFR-1 on pericytes acts as a regulator of tip cell formation in mice retinas and inhibiting this signal causes overgrowth of the vascular field (133). A full discussion of the mechanobiological roles of pericytes is beyond the scope of this review but can be found in a recent publication by Dessales et al. (5). In the context of the tumor microenvironment, CAFs are a mechanically-active stromal cell that promotes enhanced angiogenesis through growth factor signaling, matrix remodeling, and contractile behaviors (20, 76, 77, 134–138). More specifically for angiogenesis, CAFs have been shown to increase expression of growth factors including VEGF-A and stromal cell-derived factor 1 (SDF-1) after exposure to either cyclic strains or compressive forces, leading to increased angiogenesis in *in vitro* model systems (134, 137). The activation of VEGFR-2 and dissociation from VE-cadherin is regulated by a Src-dependent process that can be induced by tensile strains on pulmonary artery ECs (20). Overall, the regulation of angiogenesis by stromal cells, including VSMCs, pericytes, and CAFs, is a highly complex process based on interacting biomechanical and biochemical cues. Careful consideration of such factors should be considered when investigating therapeutic strategies that target inhibiting or promotion vascular growth through the VEGFR-2 signaling axis.

### Models to Study VEGFR-2 Signaling

As it is highly difficult to control independent biomechanical forces in *in vivo* models, most of what we know about VEGFR-2 mechanotransduction comes from *in vitro* systems. For example, pulsatile or laminar flow can reduce inflammation and stabilize the vascular wall, while a multidirectional disturbed flow can cause inflammation and lead to atherosclerosis (61, 139–144). One study examined how treating HUVECs with VEGFR-2 inhibitor ZM323881 in non-uniform shear stress conditions caused decreased expression of adhesion molecules such as VE-cadherin; however, static conditions did not show this change (145). Inflammation and atherosclerosis are involved in various cardiovascular diseases, and identifying whether VEGFR-2 signaling is involved in this mechanotransductive pathway is an essential step in harnessing the receptor to treat disease states. Though i*n vitro* experiments allow for easier control of mechanical and chemical stimulants, researchers still attempt to incorporate animal models to ensure that all aspects of the *in vivo* system are taken into account (21, 146). As the knockout of VEGFR-2 is embryonically lethal in murine models, alternative transgenic systems including inducible knockout or Cre-Lox targeted knockout must be used to determine the role of VEGFR-2 in disease progression (147–149). Some studies instead focus on heterozygous VEGFR-2 mouse models and show altered patterns of endothelial migration during embryogenesis and decreases in angiogenesis in tumor models (149, 150). One study analyzed the effects that a Y949F VEGFR-2 mutation had on ECs in mouse aortas. Cell polarity and alignment was disrupted due to this mutation, which mimicked the *in vitro* experiment using HUVECs and VEGFR-2 inhibitors SU1498 and ZM323881 (21). In mouse models utilizing a VE-cadherin Cre-based knockdown of VEGFR-2 in endothelial cells, dramatic decreases in angiogenesis were seen in retina samples, which correlated with dysregulation of VEGFR-2 patterning in cell membranes (151). Additional animal models studying VEGFR-2 signaling in myocardial ischemia involve both mouse and porcine samples and implicate TAZ interactions with VEGFR-2 (152). While such systems provide some useful information, it can result in mosaic expression of VEGFR-2 due to inconsistent Cre expression which, in combination with incomplete control over biomechanical stimuli, generates confounding data and unclear mechanisms of action. In fact, this extremely high level of physiological complexity with limited understanding of mechanobiological factors that affect VEGFR-2 may be partially responsible for the limited efficacy of therapeutic strategies that target this pathway. To address this, a variety of *in vitro* systems, including polyacrylamide hydrogels for substrate stiffness studies and microfluidic flow systems for shear stress studies, have been useful in elucidating VEGFR-2 responses to specific and highly controllable biomechanical stimuli (77, 153–160). Many such systems have been described through this review article and have generated considerable knowledge of VEGFR-2 signaling pathways. The next generation of model systems to study VEGFR-2 mechanoregulation should work to incorporate the *in vivo* complexity with the highly controllable biomechanical parameters of *in vitro* models. There is substantial clinical evidence for mutations in or alterations of VEGFR-2 signaling in not only cancer, PAD, and ischemia, but also neurological disorders such as Alzheimer's Disease, endometrial disorders, retinal degeneration, and bleeding disorders due to arteriovenous malformations (160–163). While these studies do not directly demonstrate mechanobiological regulation of VEGFR-2, there is substantial and increasing evidence that biomechanical forces are involved in all of these pathologies (3, 164–166). Therefore, a more fundamental understanding of VEGFR-2 mechanosignaling provides context and elucidation for a wide variety of disease states that may offer potential therapeutic avenues.

## Signaling

The role of biomechanical forces as regulators of cell and tissue behaviors is appreciated across many fields including cancer biology, stem cell differentiation, cardiovascular regulation, tissue engineering, and many other areas (1, 167–170). For example, a stiff bone-like matrix can cause mesenchymal stem cells (MSCs) to present traits similar to osteoblasts instead of like neural or muscle tissue (170). Studies like this revealed that the physical environment can affect cellular pathways and represent seminal foundations of the field of cellular mechanics. In recent years, there have been more descriptions of many proteins as mechanotransducers such as integrins, cadherins, and the components of the cytoskeleton (4). The biochemical signaling that results from cellular interpretations or responses to biomechanical cues is commonly referred to as mechanotransduction; the term mechanobiology includes the wider understanding of the communication of biomechanical features between a cell and its environment. In addition to regulating angiogenesis, VEGFR-2 can also be considered a mechanoreceptor, which is activated in ECs by forces such as shear stress and cyclic stretch, and this mechanoactivation causes activation of various downstream mechanotransductive signaling pathways (15, 20, 21). Through these mechanical signals, VEGFR-2 can promote angiogenesis without its VEGF ligand (76, 77). This section of the review will describe how VEGFR-2 acts as a mechanoreceptor and closely interacts with other well-characterized mechanotransductive factors, demonstrating that altered physical forces caused by disease states could affect angiogenesis. A summary of specific pathways, inhibitors, and the resulting effects of vascular growth can be found in Table 1.

**Table 1:** Effects of inhibitors on EC mechanobiological pathways.

**Protein/**	**Inhibitor**	**Effect**	**References**
**Pathway**			
ERK	PD98059	• Decreased EC proliferation on stiff and compliant matrices • Decreased EC proliferation caused by VEGF	(19)
Rho	P190RhoGAP	• P190RhoGAP knockdown results in increased *VEGFR-2* mRNA	(146)
ROCK	Y-27632	• Increases VEGFR-2 expression on EC cell membrane and decreased activation • Similar VEGFR-2 expression between ECs on 10kPa matrix treated with Y-27632 and ECs without inhibitor on 1kPa matrix	(19)
Src	PP2	• Decreased VEGFR-2, Akt, and eNOS phosphorylation caused by laminar flow	(15)
VEGFR-2	SU5416	• Increased apoptosis in osteoblasts exposed to fluid flow • Decreased ERK phosphorylation in osteoblasts exposed to fluid flow	(18)
VEGFR-2	VTI	• Decreased EC Akt and eNOS activation caused by laminar flow	(15)
VEGFR-2	SU1498	• Limited EC elongation • Lower shear stress alignment in absence of VEGF • Decreased EC Akt and eNOS activation caused by laminar flow	(15, 21)
VEGFR-2	ZM323881	• Limited EC elongation • Lower shear stress alignment in absence of VEGF • Increased shear stress alignment in presence of VEGF • Decreased adhesion molecules in ECs in non-uniform shear stress	(21, 145)
YAP	Verteporfin	• Increased DLL4 expression in ECs on a 25 kPa gel	(83)

### Receptor-Ligand Interactions

Several different isoforms of VEGF-A are produced by alternative splicing, with some of the most well-studied being VEGF121, VEGF165, VEGF189, and VEGF206; this growth factor can be secreted by many cell types including macrophages, tumor cells, and fibroblasts (23, 171). The lowest molecular weight isoform, VEGF121, is the most soluble and easily able to diffuse throughout the ECM. However, other heavier isoforms such as VEGF189 and VEGF206 remain bound to the ECM, with matrix-bound VEGF165, VEGF189, and VEGF206 all able to promote EC proliferation (172, 173). Studies have shown that the manner of ligand presentation to a receptor, either as a soluble cue or bound to the matrix, affects cell pathways and activity (174). For example, bone morphogenic protein (BMP)-2 can be soluble or matrix-bound, and cells grown on a stiff film showed slower internalization of bound BMP-2 compared to soluble (175). Binding a ligand to a matrix alters the interaction with a receptor, which in turn changes cell response, so the different presentations of VEGF could cause variable mechanoactivation of VEGFR-2. The most expressed isoform of VEGF-A is VEGF165, which can be found either in soluble form or bound to the ECM via proteoglycans (172, 173). Chen et al. studied this phenomenon by comparing VEGFR-2 phosphorylation and signaling caused by soluble (Vs) or matrix-bound (Vb) VEGF165 (16). To make Vb, VEGF was incorporated into a collagen gel. When analyzing VEGFR-2 phosphorylation as a whole, the receptor showed more sustained phosphorylation when activated by Vb than Vs. Upon studying individual VEGFR-2 tyrosine residues, researchers identified Y1214 to be the cause of this extended activation; it maintained phosphorylation for 15 min only when presented with Vb, compared to 5 min by Vs. The Y1214 phosphorylation site is associated with the ERK1/2 pathway, which is a well-known mechanosensitive pathway. This association reinforces the conclusion that VEGFR-2 phosphorylation, especially at Y1214, and activation are affected by mechanical forces within the environment (33). Further study of mechanoactivation of VEGFR-2 and the subsequent signaling cascades would provide a deeper understanding of VEGFR-2 mechanoregulation that could potentially identify novel targets for anti-angiogenic therapies in cancer treatments.

### ERK/MAPK Pathway

A well-characterized mechanosignaling pathway is the mitogen activated protein kinase, or MAPK, pathway which involves numerous kinases including the extracellular-signal-regulated kinase (ERK) (176, 177). This pathway has been implicated in several diseases including multiple types of cancers and cardiovascular diseases (178, 179). After receiving a signal from a membrane bound receptor, the MAPK pathway activates when RAS, a GTPase, activates a phosphorylation cascade involving three kinases RAF, MEK, and ERK (180). The MAPK pathway further regulates various transcription factors, including but not limited to HSP27, p38, and c-Myc, which control cell activities such as proliferation, differentiation, and migration (177, 181). ERK is a known mechanotransductive factor, translating biomechanical or biophysical signals external to the cell into a biochemical signal that controls cell response (15, 182, 183). For example, stretching or tensile strain can cause ERK activation, which leads to Madin Darby canine kidney epithelial cell (MDCK) contraction, propagation of the ERK signal in neighboring cells, and collective cell migration (184). Furthermore, ERK works with Piezo1, another known mechanoreceptor, to cause proliferation when MDCKs are stretched and apoptosis when cells are heavily confluent (185).

Several studies have demonstrated a link between VEGFR-2 and the ERK pathway, where VEGFR-2 acts upstream of ERK phosphorylation (186–188). Activation of the ERK pathway is one of the major targets of VEGFR-2 activation, and this link between ERK and VEGFR-2 is necessary for adipose mesenchymal stem cell (AMSC) to EC differentiation (186, 187). One study demonstrated that shear stress upon an EC is sufficient for VEGFR-2 phosphorylation and subsequent ERK upregulation, without the biochemical stimulation of the VEGF ligand (17). Specifically, phosphorylation of VEGFR-2 at Y1175 and Y1214 are linked to ERK activation (16, 33, 45, 189). In a study by LaValley et al., matrix stiffness was shown to be connected to both VEGFR-2 and ERK activity (19). Plating human umbilical vein endothelial cells (HUVECs) on 1 or 10 kPa collagen-coated gels showed that stiffer matrices increased the levels of phosphorylated VEGFR-2 and ERK in sub-confluent cultures, consequently increasing proliferation (175). Combining VEGF with the 10 kPa gel resulted in the highest cell growth, and the addition of the ERK inhibitor PD98059 decreased this growth. Increased matrix stiffness also promotes VEGFR-2 internalization, which then causes ERK phosphorylation and cell proliferation (19). Another study linked VEGFR-2 and ERK with mechanoactivation using pulsatile flow. In osteocytes, pulsatile fluid flow can prevent cells from undergoing apoptosis; this flow also causes an increase in ERK phosphorylation. However, when treated with the VEGFR-2 inhibitor SU5416, the osteocytes displayed increased apoptosis and decreased ERK phosphorylation (18). The link between ERK mechanotransduction and VEGFR-2 activation indicates that the receptor plays a previously underappreciated role in cellular responses to various mechanical factors. While the MAPK pathway is ubiquitous in numerous cell types, including both healthy and diseased tissues, the specificity of VEGFR-2 as a mechanoreceptor during angiogenesis could provide a unique target for promotion or inhibition of angiogenic vessel growth during disease progression.

### c-SRC Pathway

Another mechanotransductive protein downstream of VEGFR-2 signaling is Src, known as c-Src in humans, which is a cytoplasmic tyrosine kinase associated with the cell membrane or endosomal membranes. The Src protein contains Src homology (SH) domains and a kinase domain (190, 191). The role of Src has been described in many cell processes including adhesion, motility, proliferation, and differentiation, spanning across embryological development, healthy tissue homeostasis, and several disease pathways (192–198). Furthermore, Src activity is known to be at least partially regulated by mechanical cues such as shear stress and matrix adhesion, and it resides upstream of other effectors such as the ERK pathway (199, 200). In addition, Src serves as a key regulator of transforming growth factor beta (TGFβ) signaling in contractility responses of valvular interstitial cells during heart valve disease (197). These studies demonstrate the role of Src as a key intersection in numerous mechanotransductive pathways.

Moreover, Src has been shown to phosphorylate tyrosine residues on VEGFR-2, ultimately generating a positive feedback loop with further activation of both VEGFR-2 and Src (201). In one study, ECs were exposed to laminar flow in a cone and plate viscometer, which caused VEGFR-2 phosphorylation even when cells were treated with a VEGF inhibitor. However, exposure to Src inhibitor PP2 counteracted this effect and caused a decrease in phosphorylation levels of VEGFR-2 (15). This suggests that mechanical forces via shear stress cause Src to activate VEGFR-2, which could further support VEGFR-2 as a mechanoreceptor. In another study, aortic ECs from mice were studied to elucidate how VEGFR-2 and Src regulate cell alignment and polarity with respect to shear flow. Mice with a VEGFR-2 mutation Y949F, which is Y951 in humans, presented altered alignment and polarity, while mice with an inducible Src knockout in endothelial cells, only had impaired polarity (21). This reaffirms that Y951 of VEGFR-2 is linked to Src activity and also suggests that VEGFR-2 and Src interact to respond to the physical environment to control cellular polarity. Cell polarity is a necessary aspect of cell migration, development, and tissue organization; impairment of cellular polarity regulation leads to disease states including cystic kidney disease and birth defects such as neural tube defects (202–204). The role of Src as a mechanotransductive factor in the regulation of activation levels of VEGFR-2 suggest this mechanosignaling axis plays a role in vascular growth and could be targeted in novel therapeutic strategies.

### Rho/ROCK Pathway

Another well-studied protein that has been linked to both mechanoactivation of cells and VEGFR-2 is Rho. This is a GTPase that causes downstream activation of Rho-associated coiled-coil containing protein kinase (ROCK), which is a critical regulator of cytoskeletal components involved in cell migration and is typically referred to as the Rho/ROCK pathway (10, 205–210). Rho can be activated by a variety of receptors and cytokines, and ROCK is activated when phosphorylated by Rho, causing ROCK to undergo a conformation change that increases its kinase activity (205, 211). This pathway is involved in cytoskeleton regulation, fibronectin matrix control, differentiation, and apoptosis (212–215). Several studies have identified a connection between physical forces and Rho activation (10, 214, 216–218). When rat embryonic fibroblasts were subjected to mechanical strain, Rho activity, as measured by Rho being bound to GTP instead of GDP, increased compared to non-strain controls (219). A different study observed the effect of strain on capillary ECs in tumor and control environments, and the addition of strain upregulated Rho activity in the control environment but not in the tumor. Without strain, both Rho and ROCK were upregulated in tumor ECs compared to control, and since there was no significant difference between Rho activity in the strained control conditions and the tumor ECs, mechanical factors in the TME may be causing activation of the Rho/ROCK pathway (220). In another study, ECs exposed to shear stress showed a downregulation of Rho activity due to integrin binding to the ECM, which allowed the ECs to reorganize their cytoskeletal elements and align with the flow (221). Together with Src and integrins, a substantial body of work has been completed on the Rho/ROCK pathway to describe mechanotransduction in a wide variety of cells in both physiological and pathological conditions (222). Mechanical forces can work with other receptors and enzymes to increase or decrease Rho/ROCK activity in order to guide the cell cycle, differentiation, and migration.

In addition, VEGFR-2 signaling has been shown to lead to Rho/ROCK activation which then affects downstream enzymes such as focal adhesion kinase (FAK) and transcription factors such as signal transducer and activator of transcription (STAT) (30, 223, 224). One study focused on Rho inhibitor p190RhoGAP, which is naturally synthesized in cells, to see how decreases in Rho signaling altered VEGFR-2 expression and if VEGFR-2 can be mechanically-activated (146). The pseudoenzyme p190RhoGAP contains pseudo GTPase domains and can act as a Rho regulator (223). Knockdown of 190RhoGAP in ECs using siRNA caused an increase of *VEGFR-2* mRNA levels compared to the control. Additionally, *VEGFR-2* mRNA was shown to be expressed at higher levels on 4,000 Pa gels compared to a 150 Pa gel. When treated with p190RhoGAP siRNA, the 150 Pa sample *VEGFR-2* mRNA levels increased (146). This Rho inhibitor controlled VEGFR-2 levels according to the substrate stiffness used for cell culture. HUVECs grown on a stiffer gel of 10 kPa showed more VEGFR-2 endocytosis, a measure of activation, compared to HUVECs grown on a more compliant matrix of 1 kPa (19). Cells grown on a 10 kPa gel and treated with a ROCK inhibitor Y-27632 showed more VEGFR-2 on the cell membrane, suggesting that there was less activation and uptake of the receptor. The amount of VEGFR-2 present was similar to HUVECs grown on the 1 kPa gel without Y-27632. This experiment shows that the Rho/ROCK pathway is involved in regulation of VEGFR-2 endocytosis and subsequent activation in response to matrix stiffness. Understanding this complex relationship will help researchers to identify targets that could help control angiogenic activity.

### Hippo Pathway

The Hippo pathway involves a kinase cascade leading to downstream transcription factors YAP and TAZ, which are involved in organ development as well as cell contractility, migration, and proliferation (225). This pathway has thus been connected to both regenerative medicine, because of its role in development and tissue growth, and cancer, because of its influence on proliferation and cell survival (225, 226). When phosphorylated due to Hippo pathway activation, YAP and TAZ become inactive; however, VEGF promotes the opposite effect and induces YAP/TAZ activity and translocation to the nucleus (151, 227, 228). These transcription factors are key mediators of angiogenesis, and mice with an endothelial knockout of these proteins resulted in major vascular dysregulation in development (151). YAP, TAZ, and VEGFR-2 have also been linked with other angiogenic factors such as BMPs, which have shown changes in expression after ischemic conditions (152). Mechanical cues influence YAP and TAZ activity, though these effects have been shown to be at least partially independent of the Hippo pathway (225). Mammary epithelial cells (MECs) grown on stiffer matrices of 15–40 kPa show higher nuclear localization of these factors than cells grown on compliant matrices of 0.7–1 kPa, resulting in increased proliferation and decreased apoptosis (229). However, cells with a YAP/TAZ knockdown acted similarly to those grown on the compliant matrices. Since YAP/TAZ are known mechanotransducers, they likely play a role with VEGFR-2 in angiogenic response to mechanical forces. One study demonstrated that HUVECs grown on a 1 kPa matrix caused downregulation of YAP activity and upregulation of VEGFR-2 transcription levels (83). When VEGF was added, VEGFR-2 levels increased on the 1 kPa gels. YAP activation by lysophosphatidic acid causes increased delta-like ligand 4 (DLL4) expression, which is a Notch1 ligand (230). However, in HUVECs, DLL4 showed the highest expression on 1 kPa gels when YAP is least active, and addition of YAP inhibitor verteporfin also caused increased DLL4 expression in HUVECs plated on a stiff 25 kPa gel (83). Even though previous work has shown that YAP activity can promote DLL4 expression, different combinations of matrix stiffnesses and soluble VEGF leads to varied results, exemplifying how complicated mechanobiology can be. Biomechanical and biochemical signaling involves complex pathway crosstalk which needs further study in order to determine how YAP/TAZ, VEGFRs, and other angiogenic factors regulate blood vessel growth.

### Crosstalk With Other Mechanoignaling Pathways

The VEGFR-2 pathway interacts with several known important mechanotransduction pathways, as previously discussed (21, 61, 139–146). However, the crosstalk of mechanobiological agents is not exclusive to downstream signaling targets in this context. Considered as primary mechanosensors on ECs, signaling through both integrins and cell-cell adhesion molecules can lead to increases in expression of mechanotransductive factors including ERK, Src, and PI3K/Akt, among others (231–235). The interaction of adherens junctions and VEGFR-2 is required to drive responses to shear stress in ECs (22). Moreover, it has been shown that VEGF stimulation also promotes enhanced growth factor secretion in ECs including TGF-β1 and connective tissue growth factor (CTGF); inhibition of this process led to decreased basement membrane thicknesses in mouse retains (236). E-cadherin stimulation can cause increase in epidermal growth factor signaling which in turns activates the PI3K pathway (232, 237). It has also been shown that growth factor signaling can in and of itself be regulated through mechanical strain, in that activation of TGF-β1 from its latent form is increased when cells are treated with tensile forces (238, 239). Due to these confounding effects, precise model systems are needed to elucidate independent roles and reactions of VEGFR-2 in mechanosignaling.

## Anti-VEGF Treatments, Side Effects, and Efficacy

Current clinically available anti-VEGF therapeutics are either derived from antibodies or are small molecule inhibitors of the tyrosine kinase receptors, including VEGFR-2 that VEGF interacts with to promote angiogenesis. The first such therapies were available in the early 2000s, with bevacizumab approved in 2004 for colorectal cancer which was soon expanded to other types of cancer treatment including lung, glioblastoma multiforme, ovarian, renal, and metastatic breast cancer (240, 241). Other drugs approved by the USA-FDA include ranibizumab, sunitinib and sorafenib, which also have been expanded from anti-angiogenic therapies in cancers to treat a variety of cardiovascular diseases including PAD and ischemia (242). For anti-cancer treatments, the efficacy of anti-VEGF-based therapies is substantially less in clinical applications compared to preclinical *in vitro* and *in vivo* models; for example, disease free progression of breast cancer is not significantly increased when bevacizumab is delivered with other first line chemotherapeutics (240, 241, 243, 244). Based on several studies with these limited benefits, the FDA revoked approval for bevacizumab for breast cancer treatments (245). For cardiovascular diseases, such as PAD, anti-VEGF treatment strategies show little clinical improvements, despite promising preclinical work in limb ischemia models (242). Finally, in ocular vascular degenerative disorders, several anti-VEGF therapies have been approved for clinical use but show limited improvements in many clinical trials (246). While some benefits have been observed in several studies for the various diseases and conditions discussed here, the side effects associated with anti-VEGF or anti-VEGFR-2 therapies can be severe and include heightened risk of arterial thromboembolic events, hypothyroidism, wound healing complications, GI perforations, neutropenia and hematological effects and increased risks for other cardiac adverse events (245, 247–249). If future treatments combine anti-VEGFR-2 and anti-mechanotransductive elements, additional side effects could be expected if the latter pathways are ubiquitous in healthy and diseased tissues. An example of this is fasudil, a selective ROCK inhibitor that has been used to treat pulmonary hypertension, ALS, subarachnoid hemorrhages, dementia, stroke, and atherosclerosis (250–258). While long term side effects of fasudil treatment are not known, it is expected that the inhibitor will affect the cardiovascular system and potentially lead to adverse cardiac events or hypertension; one study suggested that side effects in a clinical trial were mild-moderate and ranged from skin rashes to bleeding disorders (252). On the other hand, vascular normalization therapies, where increases in VEGF signaling would promote blood vessel growth or enhanced function, demonstrate their own challenges most typically related to delivery kinetics, administration routes, and ability to evaluate clinical outcomes (259–261). To address these challenges, some groups have begun investigating gene therapy as a technique to create a more sustained and controllable deliver of VEGF; an example of this is recent data from a clinical trial where VEGF-D delivered by adeno-associated viruses decreased angina associated symptoms in a majority of patients, with similar levels of major cardiac adverse events compared to control groups (262).

## Discussion

Studies have observed complicated interactions between the biomechanical and biochemical environment which can prompt blood vessels to undergo angiogenesis or degrade functioning of existing vessels. LaValley et al. showed that individual effects of VEGF addition and a stiffer gel on cell proliferation was significantly less than when the two factors were combined (19). Understanding only the independent biomechanical or biochemical aspects of cellular signaling does not provide a full view of developmental processes or disease progression; both biochemical and biomechanical features must be studied to acquire a complete view of cellular regulation and potential novel treatment strategies.

All the diseases discussed in this paper cause both biochemical and physical changes in the matrix surrounding the cells and blood vessels, which alters normal function. For example, the cardiovascular diseases mentioned—PAD, ischemia, arterial stiffening—result in some form of matrix stiffening. In addition, the TME contains higher levels of collagen, while arterial stiffening, which is common in PAD and ischemia, is a result of less elastin and more collagen (6, 8). The vascular ECs in these diseases are surrounded by a stiffer matrix, and studies have shown less angiogenic sprouting, increased VEGFR-2 activation, and more cell growth in stiffer gels (19, 75). Additionally, the abnormal vasculature in tumors and the atherosclerosis in cardiovascular diseases both can cause abnormal shear stress within the vessels (2, 90). Patients that experience claudication often exercise less which causes lower fluid shear stress, and regular intervals of high shear stress is known to promote healthy vasculature (263). Flow regulates various cell activities such as the phosphorylation of proteins like VEGFR-2 and ERK and the orientation and polarity of the cell body (15, 18, 21). Because of mechanical changes present in disease states and in a developing embryo, further understanding of the relationship between cytokines, physical forces, and mechanotransducers will help researchers to determine how to either inhibit or promote angiogenesis to treat diseases or develop improved vascularization strategies for tissue engineering.

## Future Directions

Regarding VEGFR-2 and angiogenesis, further investigation is required to understand how different types of physical forces such as pressure, matrix stiffness, tensile strains, and fluid shear stress affect VEGFR-2 activation and internalization. Furthermore, investigations of different VEGFR-2 tyrosine residues and how changes in biomechanical factors cause and sustain phosphorylation would help to clarify the interactions VEGFR-2 has with other enzymes and transcription factors. Identifying the exact phosphorylation site triggered by specific biomechanical stimuli and the resulting signaling cascades would help researchers develop ways to promote or inhibit relevant signals. As mentioned previously, additional research could be directed toward the complex relationship and interaction between chemical and mechanical cues. Since the combination of the two can produce the greatest effect and act synergistically, understanding these processes would open more directions of study that could ultimately lead to better control of angiogenesis. Specifically, a rise in sophisticated microfluidic systems designed to investigate individual and independent biomechanical and biochemical signals will be crucial to fully elucidate these complex interactions (72, 77, 154, 264). Finally, there are several members of the VEGF ligand family, each with various isoforms. The most common, VEGF-A, has some isoforms that are mainly soluble and other which are mainly matrix-bound, and Chen et al. discovered a difference caused by soluble vs. matrix-bound VEGF (16). This type of prolonged and site-specific activation represents an understudied phenomenon in VEGFR-2 regulation. The relationship between the VEGF and VEGFR families is complex due to the various members and isoforms, but each plays a role that needs to be more fully understood.

## Conclusion

Mechanical forces, along with various cytokines, regulate the formation and maintenance of the vasculature. Thus, angiogenesis is a process that is central not only to development but also in many diseases. Additionally, the field of regenerative medicine should focus not only on chemical stimuli but also mechanical stimuli in order to most effectively develop new vasculature in scaffold structures. Investigations of how VEGFR-2, along with other connected pathways such as ERK/MAPK, Src, Rho/ROCK, and YAP/TAZ are mechanically controlled have demonstrated that mechanics are an essential focus in understanding how to evolve therapeutic angiogenesis or in preventing angiogenesis in the TME. Further study of VEGFR-2, its related pathways, and the interaction of biochemical and biomechanical signaling that control vascular growth will aid researchers in developing new treatments that more efficiently treat the underlying cause of various disease states.

## Author Contributions

BM wrote the first draft of the manuscript. BM and MS-L contributed to manuscript revision, read, and approved the submitted version. All authors contributed to the article and approved the submitted version.

## Funding

This work was supported by National Institutes of Health (CA230202 MS-L), O'Neal Comprehensive Cancer Center (IMPACT Award MS-L).

## Conflict of Interest

MS-L receives compensation for consulting services for CerFlux, Incorporated. The remaining author declares that the research was conducted in the absence of any commercial or financial relationships that could be construed as a potential conflict of interest.

## Publisher's Note

All claims expressed in this article are solely those of the authors and do not necessarily represent those of their affiliated organizations, or those of the publisher, the editors and the reviewers. Any product that may be evaluated in this article, or claim that may be made by its manufacturer, is not guaranteed or endorsed by the publisher.
